# PD-L1 near Infrared Photoimmunotherapy of Ovarian Cancer Model

**DOI:** 10.3390/cancers14030619

**Published:** 2022-01-26

**Authors:** Jiefu Jin, Ishwarya Sivakumar, Yelena Mironchik, Balaji Krishnamachary, Flonné Wildes, James D. Barnett, Chien-Fu Hung, Sridhar Nimmagadda, Hisataka Kobayashi, Zaver M. Bhujwalla, Marie-France Penet

**Affiliations:** 1 Division of Cancer Imaging Research, The Russell H. Morgan Department of Radiology and Radiological Science, The Johns Hopkins University School of Medicine, Baltimore, MD 21205, USA; jjin@jhmi.edu (J.J.); isivaku3@jhmi.edu (I.S.); ymironc1@jhmi.edu (Y.M.); bkrishn1@jhmi.edu (B.K.); fwildes1@jhmi.edu (F.W.); jbarne55@jhu.edu (J.D.B.); zbhujwa1@jhmi.edu (Z.M.B.); 2 Sidney Kimmel Comprehensive Cancer Center, The Johns Hopkins University School of Medicine, Baltimore, MD 21205, USA; chung2@jhmi.edu (C.-F.H.); snimmag1@jhmi.edu (S.N.); 3 Division of Nuclear Medicine and Molecular Imaging, The Russell H. Morgan Department of Radiology and Radiological Science, The Johns Hopkins University School of Medicine, Baltimore, MD 21205, USA; 4 Molecular Imaging Program, Center for Cancer Research, National Cancer Institute, US National Institutes of Health, Bethesda, MD 20814, USA; kobayash@mail.nih.gov; 5 Department of Radiation Oncology and Molecular Radiation Sciences, The Johns Hopkins University School of Medicine, Baltimore, MD 21205, USA

**Keywords:** ovarian cancer, photoimmunotherapy, theranostics

## Abstract

**Simple Summary:**

Ovarian cancer is one of the leading causes of cancer-related death among women in the United States. Overall survival of patients with advanced stage disease has not significantly changed despite improvements in treatment that have extended median survival. Photoimmunotherapy (PIT) using an antibody conjugated to a near infrared (NIR) dye constitutes an effective theranostic strategy to detect and selectively eliminate cell populations. The programmed death-1 (PD-1)/programmed death ligand-1 (PD-L1) signaling pathway is being extensively studied for immune checkpoint blockade. PD-L1 expression has been described across all major ovarian cancer histological subtypes and is commonly expressed by cancer cells and by tumor-associated macrophages (TAMs). Here, we used NIR-PIT to eliminate PD-L1-expressing TAMs and cancer cells in ovarian cancer xenografts. Overall, our findings support using NIR-PIT as a potential therapeutic option for ovarian cancer.

**Abstract:**

(1) Background: Despite advances in surgical approaches and drug development, ovarian cancer is still a leading cause of death from gynecological malignancies. Patients diagnosed with late-stage disease are treated with aggressive surgical resection and chemotherapy, but recurrence with resistant disease is often observed following treatment. There is a critical need for effective therapy for late-stage ovarian cancer. Photoimmunotherapy (PIT), using an antibody conjugated to a near infrared (NIR) dye, constitutes an effective theranostic strategy to detect and selectively eliminate targeted cell populations. (2) Methods: Here, we are targeting program death ligand 1 (PD-L1) using NIR-PIT in a syngeneic mouse model of ovarian cancer. PD-L1 PIT-mediated cytotoxicity was quantified in RAW264.7 macrophages and ID8-Defb29-VEGF cells in culture, and in vivo with orthotopic ID8-Defb29-VEGF tumors. (3) Results: Treatment efficacy was observed both in vitro and in vivo. (4) Conclusions: Our data highlight the need for further investigations to assess the potential of using NIR-PIT for ovarian cancer therapy to improve the treatment outcome of ovarian cancer.

## 1. Introduction

Ovarian cancer is one of the leading causes of cancer-related deaths among women in the United States [1,2]. The 90% five-year survival rate at early diagnosis drops precipitously to 27% when women are diagnosed at stage III or IV, as by that time aggressive metastases have already formed in adjacent abdominal organs. High morbidity and mortality are caused by advanced stage disease presentation that lacks effective therapy [3]. Overall survival of patients with advanced stage disease has not significantly changed despite improvements in treatment that have extended median survival [4]. The initial treatment for patients with advanced stage disease consists of extensive debulking surgery. Improved survival is observed after optimal surgical debulking, with less than 1 cm of residual disease, as compared to bulky residual disease, with more than 1 cm of residual disease. After surgery, patients undergo a combination of platinum–taxane chemotherapy [4]. While cytoreductive surgery followed by combination chemotherapy have remained standard of care for decades, novel targeted therapies are being incorporated when appropriate. These therapies include poly(ADP-ribose) polymerase (PARP) inhibitors, the anti-VEGF antibody bevacizumab, or more recently, treatment with immune checkpoint inhibitors [3]. Most advanced stage ovarian cancer patients eventually suffer recurrence and develop drug-resistant tumors. The lack of curative treatment and the high rate of relapse for women with advanced stage ovarian cancer create an urgent unmet need for new therapeutic strategies.

Photoimmunotherapy (PIT) provides an effective theranostic strategy to detect and selectively eliminate cells by conjugating a near infrared (NIR) dye to an antibody. Following light irradiation, NIR-PIT selectively damages cells to which the antibody is attached, while also activating the local host immune system, leading to longer lasting anti-tumor response [5]. Highly specific necrotic and immunogenic cell death can be induced by NIR-PIT in tumors, with only minimal adverse effects in normal tissue [6]. After binding of the antibody-IR700 conjugate, subsequent local exposure to NIR light induces physical changes in the structure of the antibody–antigen complexes that are thought to induce physical stress within the cellular membrane. This leads to increased transmembrane water flow that eventually leads to the cell rupturing. Cell death results in the proliferation of CD8 + T cells against a variety of released cancer antigens, which amplifies the therapeutic effect of NIR-PIT [7]. The efficacy of NIR-PIT has been shown in multiple preclinical tumor models [6,8,9,10]. 

A first-in-human phase I/II clinical trial of NIR-PIT targeting epithelial growth factor receptor (EGFR) in patients with inoperable head and neck squamous cell cancer was successfully concluded in late 2017 (NCT02422979). The study used RM-1929, combining an IR700 dye with Erbitux^®^ (cetuximab), an FDA-approved antibody that targets EGFR, highly expressed in squamous cell carcinomas of the head and neck (HNSCC). The phase I dose escalation study demonstrated safety and tolerability of RM1929-PIT. Improvements for patients with recurrent HNSCC were observed. No dose-limiting toxicities were described, and no photosensitivity reactions were observed. Some patients experienced serious adverse effects, including oral pain, tumor hemorrhage, and tumor pain [11]. Safety was also described in a Phase I study in Japan [12].

In the phase IIa study, non-thermal red light was applied to the tumors 24 h post infusion of RM-1929. The light was applied either by surface illumination for superficial disease or interstitial illumination via intratumoral placement of fiber optic diffusers for deep tumors [11,13,14]. No dose-limiting toxicities or skin photosensitivity reactions were observed. Serious adverse events (SAEs) reported included treatment site pain, tumor hemorrhage, and swelling. RM-1929 PIT treatment was generally well tolerated with the majority of AEs classed as mild to moderate in severity. Responses and survival following RM-1929 PIT treatment warrant further investigation. 

The phase IIa results showed an unconfirmed objective response rate of 43.3% and a confirmed objective response rate of 26.7% in recurrent HNSCC patients who had failed standard of care. RM-1929 PIT was associated with longer median overall survival (9.3 months), with four patients achieving complete responses (13%) [11].

These early results suggest that NIR-PIT is superior to existing second- and third-line therapies for recurrent head and neck cancers [15]. 

A “fast-tracked” global phase III clinical trial began in 2019 (NCT03769506) to evaluate the efficacy and safety of ASP-1929 PIT for the treatment of locoregional, recurrent HNSCC in patients who have failed or progressed on or after at least two lines of therapy. RM-1929 and ASP-1929 are analogous, with comparable physical and chemical properties. 

The study will include ~275 subjects in a 2:1 randomization (ASP-1929 PIT:Physician’s choice SOC). The physician’s choice SOC arm includes cetuximab, methotrexate, or docetaxel.

NIR-PIT was approved for clinical use in Japan in September 2020 for recurrent head and neck cancer patients. Since January 2021, the therapy has been under health insurance coverage in 15 hospitals, and is now performed in 40 Japanese hospitals. 

The high degree of spatial and temporal control of cytotoxicity associated with NIR-PIT makes it a good candidate to treat localized and disseminated disease often observed in ovarian cancer patients [16]. 

The programmed death-1 (PD-1)/programmed death ligand-1 (PD-L1) signaling pathway is being extensively studied in ovarian cancer. PD-1/PD-L1 pathway inhibitors used as monotherapy in ovarian cancer have shown lower response rates as compared to other cancer types [17]. The ovarian cancer tumor microenvironment is characterized by immune suppression and tolerance [18]. One of the crucial mechanisms of immune surveillance escape is the up-regulation of immune checkpoints blocking activated cytotoxic T cells. New therapeutic strategies to activate the immune-suppressive ovarian cancer immune microenvironment, such as targeting PD-L1, are urgently required. PD-L1 expression has been described across all major ovarian cancer histological subtypes and is commonly expressed by cancer cells and tumor-associated macrophages (TAMs) [19,20]. In ovarian cancer, TAMs are particularly abundant among the immune cells recruited to the tumor, and are associated with tumor progression, angiogenesis, metastases, ascitic fluid build-up, and immune suppression [21]. 

An open-label study of ASP-1929 PIT combined with anti-PD1 therapy was recently started in EGFR-expressing advanced solid tumors (NCT04305795). In this study, patients with recurrent or metastatic squamous cell cancer of the head and neck, or advanced or metastatic cutaneous squamous cell carcinoma will receive anti-PD1 therapy with an anti-EGFR antibody–dye conjugate, ASP-1929, followed by PIT. 

Here, we applied NIR-PIT to target PD-L1 in a syngeneic orthotopic model of ovarian cancer. NIR-PIT efficacy was first assessed in ovarian cancer cells and macrophages, followed by in vivo studies in a syngeneic orthotopic model of ovarian cancer. Assessment of the effects of PD-L1 PIT requires the use of a syngeneic tumor model with an intact immune system. Only limited murine ovarian cancer models are available. ID8 ovarian cancer cells originated from ovarian surface epithelial cells in an immune competent C57/BL6 mouse [22]. The ID8 cell line was further transformed to overexpress VEGF [23], and beta-defensin was added to the model to interact with VEGF, and further increase tumor vascularization [24]. This modified cell line grows well orthotopically [25], and allows investigation into the effects of NIR-PIT on tumor progression in ovarian cancer in mice with an intact immune system. We also measured the efficacy of targeting F4/80 and CD11b expressed by macrophages in vitro. F4/80 is a widely used marker of murine macrophage populations, while CD11b is expressed on the surface of many leukocytes, including monocytes, granulocytes, macrophages, and natural killer cells.

## 2. Materials and Methods

Synthesis and characterization of IR700-conjugated anti-PD-L1, CD11b, F4/80, and IgG monoclonal antibodies: The water soluble phthalocyanine dye, IRDye 700DX NHS ester (IR700) was obtained from Li-Cor Bioscience (Lincoln, NE, USA). Anti-PD-L1 monoclonal 10F.9G2 IgG2b antibody, anti-CD11b monoclonal rat IgG2b antibody (clone: M1/70), anti-F4/80 monoclonal rat IgG2b antibody (clone: CI:A3-1), and rat IgG2b were obtained from BioXCell (Lebanon, NH, USA). In a typical antibody-IR700 conjugate synthesis, 1 mg of antibody was dispersed first in 1 mL of PBS and then added with 159.2 μg of IR700 (81.6 nmol, 1 mM in DMSO). The mixture was kept overnight at 4 °C, and the antibody conjugate was purified using ultra-centrifugal filter units (Millipore Amicon Ultra-0.5, 10 kDa, Billerica, MA, USA) to remove the unbound IR700 molecules. The IR700-conjugated anti-PD-L1, anti-CD11b, and anti-F4/80 monoclonal antibodies obtained were abbreviated as PD-L1-IR700, CD11b-IR700, and F4/80-IR700, respectively. Similarly, we obtained rat IgG conjugated with IR700 as a non-target isotype control, which was abbreviated as IgG-IR700. The concentration of antibody and the dye/protein ratio was determined spectroscopically by measuring the absorbance of the conjugate at 280 nm and 689 nm. The extinction coefficients were 210,000 M^−1^ cm^−1^ for all antibodies, and 165,000 M^−1^ cm^−1^ for IR700 at 689 nm. The correction factor of IR700 at 280 nm was 0.095. 

Cell culture: RAW264.7 cells were purchased from American Type Culture Collection (ATCC, Manassas, VA, USA). ID8-defb29-VEGF and MOSE (murine ovarian surface epithelial) cells, obtained from Dr. Hung, and RAW264.7 were cultured in 10% fetal bovine serum (FBS)-supplemented RPMI 1640 (Sigma, St. Louis, MO, USA), and maintained at 37 °C in a humidified atmosphere containing 5% CO_2_.

Flow cytometry analysis: ID8-Defb29-VEGF, MOSE, and RAW264.7 were seeded at a density of 1 × 10^6^ in 100 mm tissue culture dishes. Twenty-four hours later, cells were treated with 10 ng/mL of murine IFN-γ (R&D Systems, Minneapolis, MN, USA) for 24 h. The following monoclonal antibodies were used: anti-PD-L1-PE (phycoerythrin) (clone MIH5, BD Pharmingen, San Diego, CA, USA), anti-CD11b-PE (clone M1/70, Invitrogen, Waltham, MA, USA), anti-F4/80-APC (allophycocyanin) (Invitrogen, Waltham, MA, USA), and antiCD68-APC (clone FA11 Biolegend, San Diego, CA, USA). A kit for cell perforation and fixation was used for CD68, as it is intracellular. Briefly, PBS EDTA 5 mM buffer was used to wash and harvest cells. Approximately 5 × 10^5^ cells were resuspended in PBA buffer (PBS containing 0.5% bovine serum albumin and 0.02% sodium azide), incubated in the dark at 4 °C for 1 h with the different antibodies, and washed in PBA three times. Flow analyses were performed with a FACScalibur system (Becton Dickinson Immunocytometry Systems, San Jose, CA, USA). IgG-PE and IgG-APC controls (BD Pharmingen, San Diego, CA, USA) were used to delineate specific stained populations. The percentage of positive events was measured using FlowJo software (FLOWJO, LLC, Ashland, OR, USA).

qRT-PCR and Immunoblots on cell and tumor extracts: A QIAshredder and an RNeasy Mini Kit (Qiagen, Hilden, Germany) were used to isolate total RNA from cells, and an iScript cDNA synthesis Kit (Bio-Rad, Hercules, CA, USA) was used to prepare cDNA. Real-time PCR was performed using IQ SYBR Green supermix and gene-specific primers in the iCycler real-time PCR detection system (CFX-96 Connect; Bio-Rad) with cDNA samples diluted 1:10. Primers were either designed using Beacon designer software 7.8 (PREMIER Biosoft, Palo Alto, CA, USA), with Primer3Plus (opensource software), or synthesized based on previously published work (see Supplementary Table S1 for primer details). The expression of target RNA relative to the housekeeping ribosomal gene HPRT was determined based on the threshold cycle (C_t_), as ΔC_t_ = (C_t_ of target gene) − (C_t_ of 18s rRNA). To calculate fold induction, the ΔC_t_ values from all the biological replicates were averaged to obtain ave. ΔC_t_. Later, ΔΔC_t_ was calculated by subtracting ave. ΔC_t_. from ave. ΔC_t_. of individual samples. Fold induction was calculated as 2^−ΔΔCt^.

Proteins were extracted from either cells or freeze-clamped tumors to perform immunoblot analysis (the original blots see Supplementary Figure S10). RIPA lysis buffer fortified with a protease inhibitor cocktail, dithiothreitol, phenylmethylsulfonyl fluoride, sodium orthovanadate, and sodium fluoride (Sigma) was used for the extraction. A Bradford Bio-Rad protein assay Kit (Bio-Rad, Hercules, CA, USA) was used to estimate protein concentration. Then, 80 μg of total protein was resolved on 10% SDS-PAGE, transferred onto nitrocellulose membranes, and probed with a mouse specific PD-L1 (AF1019 R&D) (dilution 1:200), a mouse specific CD68 antibody (28058-1-AP Proteintech, Rosemont, IL, USA) (dilution 1:1000), a mouse specific CD11b antibody (ab133357 Abcam, Cambridge, UK) (dilution 1:2500), for 2 h at room temperature. GAPDH or β-actin was used as loading control and detected with a human and mouse cross-reactive monoclonal antibody (G8795 and AC-15, respectively, Sigma, St. Louis, MO, USA) (dilution 1:50,000). Immunoblots were developed using the SuperSignal West Pico chemiluminescent substrate Kit (Thermo Fisher Scientific, Waltham, MA, USA). 

In vitro NIR PIT-induced cytotoxicity assay: Ten thousand cells were seeded onto 96-well plates for an incubation of 24 h. Twenty-four hours later, IFN-γ treated cells received 10 ng/mL of murine IFN-γ for 24 h. For NIR-PIT experiments, the medium was replaced with the corresponding fresh medium containing the following reagents: PBS, 20 μg/mL PD-L1-IR700, CD11b-IR700, F4/80-IR700, or IgG-IR700. Cells were further incubated for 1 h at 37 °C. After washing, fresh medium was added for 30 min. A light-emitting diode (Marubeni, Santa Clara, CA, USA) provided NIR irradiation to the cells at the peak wavelength of 690 nm and irradiation at 8, 16, or 32 J/cm^2^. Post NIR irradiation, 10 μL of CCK-8 reagent (Dojindo, Rockville, MD, USA) was added to the cells in each well for a 3 h incubation at 37 °C. For controls, cells were exposed to the same incubation conditions but kept in the dark using aluminum foil to wrap the plates. The cell medium absorbance was measured at 450 nm. Cytotoxicity data were expressed as mean ± standard derivation (SD) from triplicate trials (performed in quadruplet).

Tumor implantation: Tumors were implanted orthotopically in albino C57BL/6J female mice (B6N-*Tyr^c-Brd^*/BrdCrCrl) (Charles River, Wilmington, MA, USA) using a two-step process [25,26]. Subcutaneous tumors were grown by inoculating 2 × 10^6^ ID8-Defb29-VEGFcells in 50 μL of Hanks balanced salt solution in the right flank of C57BL/6J female mice until they reached a size of approximately 100–200 mm^3^. Under sterilized conditions, the tumors were then excised, cut into ~1 mm pieces, and surgically implanted in the ovaries of anesthetized albino C57BL/6J female mice. To assess tumor progression, mice were scanned weekly starting 2 weeks post implantation. Experiments were performed when the tumors reached volumes of ~200–300 mm^3^. All surgical procedures and animal handling were performed in accordance with protocols approved by the Johns Hopkins University Institutional Animal Care and Use Committee and conformed to the Guide for the Care and Use of Laboratory Animals published by the NIH.

In vivo MRI and fluorescence imaging: All MR acquisitions were performed on a 9.4 T Bruker spectrometer. T1-weighted anatomic images were acquired using a Bruker 30 mm volume coil placed around the mouse torso. Fluorescence imaging of tumor-bearing mice was performed on a Li-Cor Pearl^®^ Impulse imager (LI-COR Biosciences, Lincoln, NE, USA).

In vivo photoimmunotherapy: Mice bearing orthotopic ID8-Defb29-VEGF tumors were randomly assigned to three groups receiving i.v. injection of PBS, 100 μg of IgG-IR700, or PD-L1-IR700, once the tumor volume reached 100 mm^3^, as measured by MRI. Twenty-four hours post injection, tumors were exposed to NIR irradiation at 200 J/cm^2^ (provided by an LED from Marubeni, Santa Clara, CA, USA) at a peak wavelength of 690 nm. The rest of the body was covered by aluminum foil. Fluorescent imaging with the Li-COR Pearl^®^ Impulse scanner was performed before and after light exposure. Tumor growth was followed weekly with MRI over a 3-week period. After three weeks of monitoring, mice were euthanized, and the tumors were excised for further analysis.

Toxicity studies: Plasma creatinine, blood urea nitrogen (BUN), bilirubin, alanine aminotransferase (ALT), and aspartate aminotransferase (AST) levels were evaluated by the Johns Hopkins University School of Medicine Phenotyping Core Facility. Plasma was obtained from ID8-Defb29-VEGF tumor-bearing mice, 12 h post light exposure, i.e., 36 h post injection of PDL1-IR700. Plasma samples collected at three weeks post treatment were also analyzed. Liver, kidney, heart, and lungs were fixed in formalin, sectioned at 5 μm thickness, and stained with hematoxylin and eosin (H&E). Lungs were inflated with agarose before formalin fixation. Percent damaged areas in the liver and lungs sections were determined by obtaining a ratio of the damaged to the total organ area in each section, using ImageJ software to analyze images acquired at 1×.

Statistical Analysis: Data presented are mean ± standard deviation (SD). Statistical analysis was performed using Prism GraphPad (San Diego, CA, USA) using a two-sided *t*-test, assuming unequal variance for comparison between two groups. Values of *p* ≤ 0.05 were considered significant.

## 3. Results

**Figure 1:**
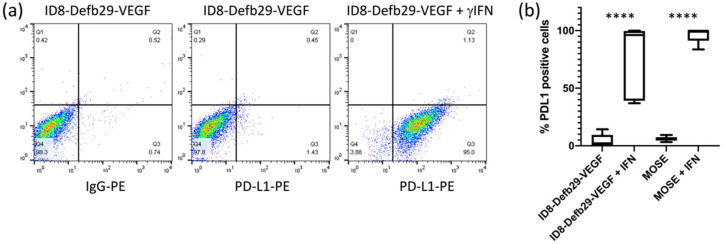
Representative flow cytometry dot plots of ID8-Defb29-VEGF cells without and with IFN-γ (**a**), and percentage of PD-L1-positive ID8-Defb29-VEGF (*n* = 10) and MOSE (*n* = 9) cells without and with IFN-γ (**** *p* < 0.0001) (**b**).

**Figure 2:**
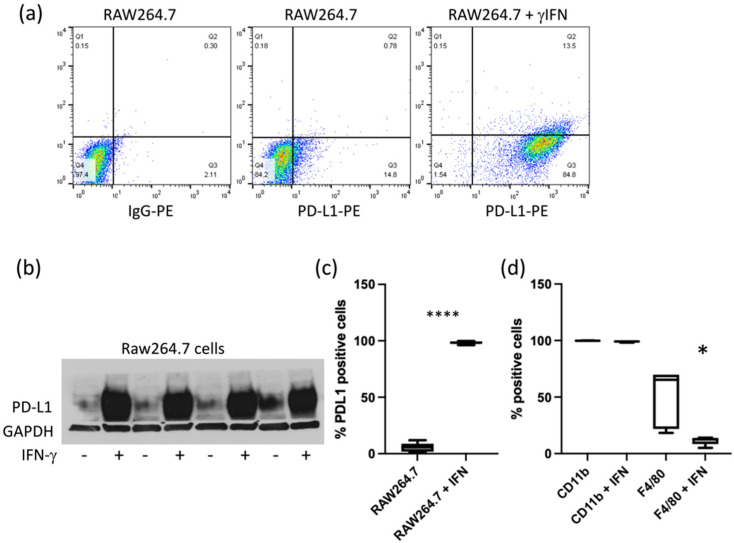
Representative dot plots of RAW264.7 cells without and with IFN-γ (**a**). Immunoblots of cell extracts showing the increase of PD-L1 with IFN-γ treatment (**b**). Percentage of PD-L1 positive RAW264.7 cells (*n* = 7 without IFN, and *n* = 10 with IFN) (**** *p* < 0.0001) (**c**). Percentage of CD11b- (*n* = 4) and F4/80- (*n* = 5) positive RAW264.7 cells with and without IFN-γ (* *p* < 0.05) (**d**).

Although the basal expression level of PD-L1 in ID8-Defb29-VEGFand MOSE cells in culture was low, it was strongly induced by IFN-γ, as shown by flow cytometry analysis (Figure 1). Similarly, PD-L1 expression in RAW264.7 cells was increased by IFN-γ (Figure 2a–c). Expression of CD11b and F4/80 was confirmed in RAW264.7 cells by flow cytometry. CD11b levels were not significantly modified by IFN-γ, but we observed a decrease in F4/80 (Figure 2d). CD68, usually used as a macrophage marker, was detected in both RAW264.7 and ID8-Defb29-VEGF cells (Figure S1a). Flow analysis results were confirmed by western blot (Figure S1b).

**Figure 3:**
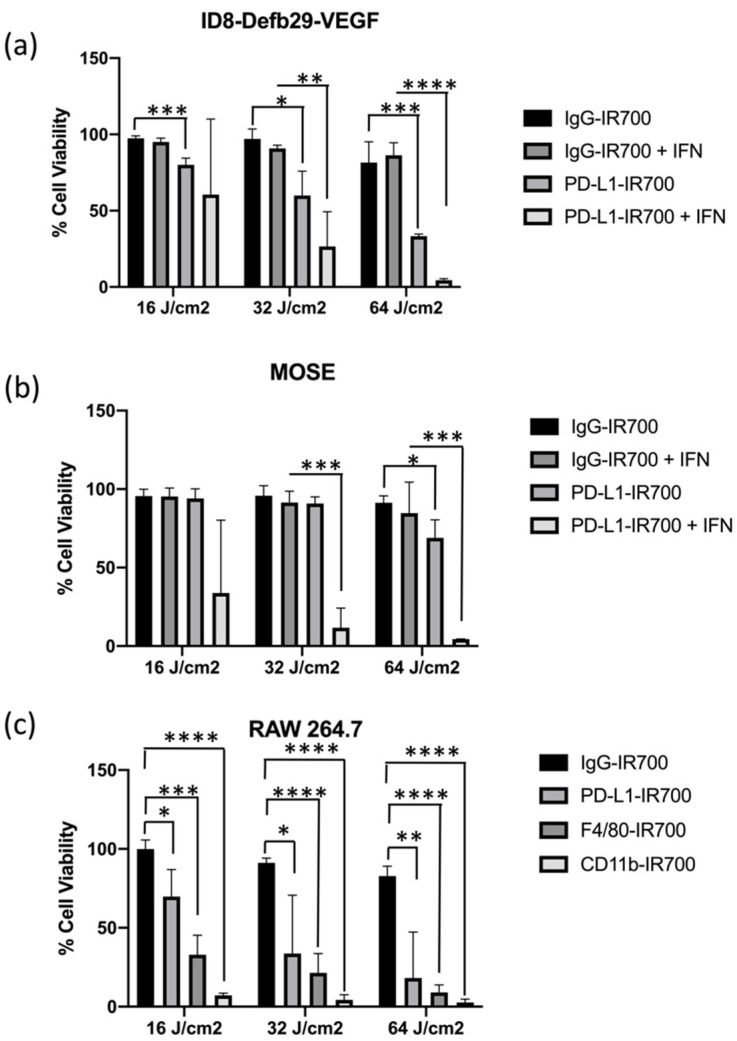
Percent cell viability following IgG-IR700 or PD-L1-IR700 NIR-PIT with increasing doses of light irradiation (16, 32, or 64 J/cm^2^) of ID8-Defb29-VEGF (**a**) and MOSE (**b**) cells with or without IFN-γ pre-treatment (*n* = 3, each with four technical replicates). Percent cell viability following IgG-IR700, PD-L1-IR700, F4/80-IR700, or CD11b-IR700 NIR-PIT with increasing doses of light irradiation (16, 32, or 64 J/cm^2^) of RAW264.7 cells (*n* = 3 (four replicates) (**c**); * *p* < 0.05; ** *p* < 0.01; *** *p* < 0.005; **** *p* < 0.001.

We performed in vitro NIR-PIT experiments targeting PD-L1 expressed by MOSE, ID8-Defb29-VEGF, and RAW264.7 cells with or without IFN-γ for 24 h to increase PD-L1 expression (Figure 3). RAW264.7 cells were treated with either PD-L1-IR700, CD11b-IR700, or F4/80-IR700. In ID8-Defb29-VEGF cells, photo-irradiation dose-dependent efficacy was observed with and without IFN-γ (Figure 3a). The efficacy of treatment was significantly higher following induction of PD-L1. Cell death in MOSE cells was observed only after treatment with IFN-γ with the two highest doses of light irradiation (Figure 3b). ID8-Defb29-VEGF cells were more sensitive to NIR-PIT treatment. Significant cell toxicity was observed without IFN-γ at the three doses tested. IgG-IR700, used as control, did not induce any toxicity, even at the highest photo-irradiation dose. Treatment with IFN-γ did not change the effects of IgG-IR700 combined with phot-irradiation in both ovarian cancer cell lines.

Dose-dependent toxicity was observed in RAW264.7 cells with the three targets tested, PD-L1, CD11b, and F4/80 (Figure 3c). The strongest effect was observed with CD11b, where the lowest photo-irradiation dose achieved maximum cell killing. This result correlates with the high expression levels of CD11b, as shown by flow cytometry and immunoblot analysis. Cytotoxicity of PD-L1-IR700 NIR-PIT was observed in the absence of IFN-γ induction. A slight cytotoxic effect was observed with IgG-IR700 at the highest irradiation dose. Pretreatment of RAW264.7 with IFN-γ improved the cytotoxicity induced by PD-L1-IR700 NIR-PIT. However, treating the cells with IFN-γ made them sensitive to treatment with IgG-IR700 NIR-PIT, which was not observed without IFN-γ pretreatment (Figure S2a). When cells were treated with PD-L1-IR700, CD11b-IR700, and F4/80-IR700 without exposure to light irradiation, no toxicity was observed (Figure S2b).

**Figure 4:**
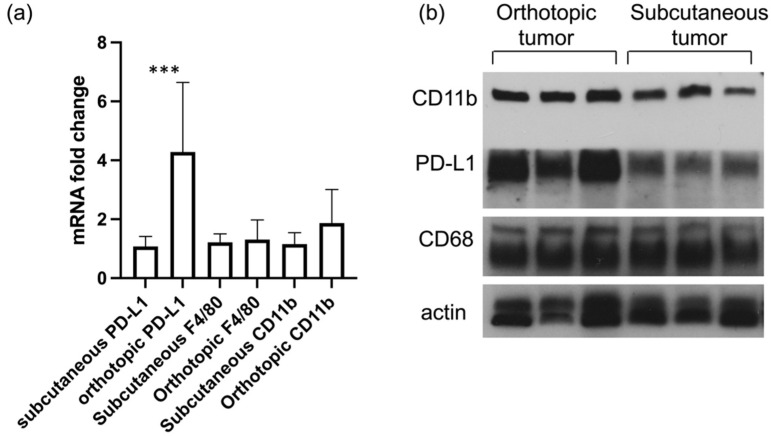
mRNA levels of PD-L1, F4/80, and CD11b in subcutaneous and orthotopic ID8-Defb29-VEGF tumors (*n* = 8, *** *p* < 0.005) (**a**). Immunoblots of three subcutaneous and three orthotopic ID8-Defb29-VEGF tumors showing expression levels of CD11b, PD-L1, and CD68 (**b**). Actin was used as loading control.

While subcutaneous models present advantages for NIR light irradiation and to follow tumor progression, orthotopic tumor models provide a more accurate model of the human disease. We analyzed the mRNA and protein levels of PD-L1 and CD11b in ID8-Defb29-VEGF subcutaneous and orthotopic tumors (Figure 4). We observed a significantly higher level of PD-L1 at the mRNA and protein levels in the orthotopic tumors as compared to the subcutaneous tumors. No significant difference in F4/80 was measured between either subcutaneous or orthotopic models.

**Figure 5:**
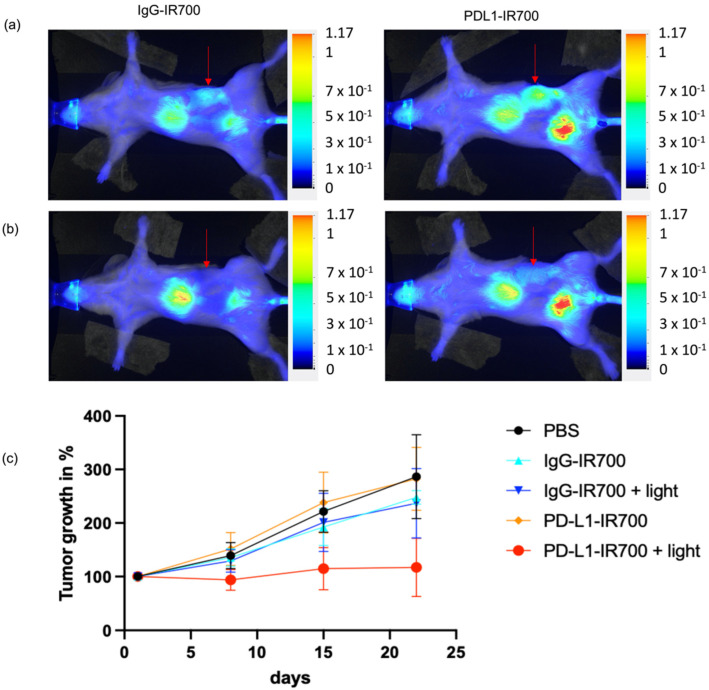
NIR images of representative ID8-Defb29-VEGF orthotopic tumor- (marked by arrow) bearing mice 24 h post injection of IgG-IR700 (left column) or PD-L1-IR700 (right column), before (**a**) and immediately after (**b**) photoirradiation. Normalized tumor growth of ID8-Defb29-VEGF orthotopic tumors treated with PBS (*n* = 5), IgG-IR700 (*n* = 3), IgG-IR700 + light (*n* = 8), PD-L1-IR700 (*n* = 5), PD-L1-IR700 + light (*n* = 8) (**c**).

Orthotopic tumor progression was followed with MRI. When tumors reached a volume of ~200 mm^3^, mice were injected with either PD-L1-IR700, IgG-IR700, or PBS. The tumor area was exposed to light 24 h post injection, with aluminum foil protecting the rest of the animal to ensure that only the tumor area was exposed to light. An example of mouse light exposure and protection with aluminum foil is presented in Supplementary Figure S3. Tumor volumes were measured on the day of injection, then weekly for a total of 3 weeks post injection. Tumor progression was reduced in the group of mice treated with PD-L1-IR700 NIR-PIT compared to the groups treated with PD-L1-IR700 without light, or with IgG-IR700 with or without light, or with PBS (Figure 5). 

Toxicity was observed in the PD-L1-IR700 NIR-PIT group; 11 mice died 24 h post light exposure. Tumor growth was followed in the nine surviving mice. 

To better understand the toxicity observed in the PD-L1-IR700 NIR-PIT group, we collected plasma, heart, kidneys, liver, and lungs from mice 12 h post light exposure (36 h post injection of PDL1-IR700). Tumor-bearing mice injected with either PBS or PDL1-IR700 were used as controls, without light exposure. The results (summarized in Supplementary Figures S3–S9) showed that lungs and kidneys were slightly damaged by exposure to light, as shown by AST, ALT, and BUN levels, and in histology sections. No differences were measured in creatinine or bilirubin. 

Out of the five mice treated with PDL1-PIT, one showed significant damage to the lungs (4.29% on IHC section) (Supplementary Figure S9). The same mouse had the highest levels of AST and ALT and a damaged liver area of 6.91% (an average of 0.79% was observed in the other four mice).

Spatial separation ensured that while the kidneys and liver can be affected directly by light irradiation, the lungs were not exposed to light. The effects observed in the lungs seem to be a consequence of the tumor bearing mouse receiving PDL1-IR700 and light that may have significantly modified the balance of immune checkpoint expression. A similar effect has been observed with multiple injection of anti-PDL1 antibodies that resulted in death only in tumor-bearing mice [27].

**Figure 6:**
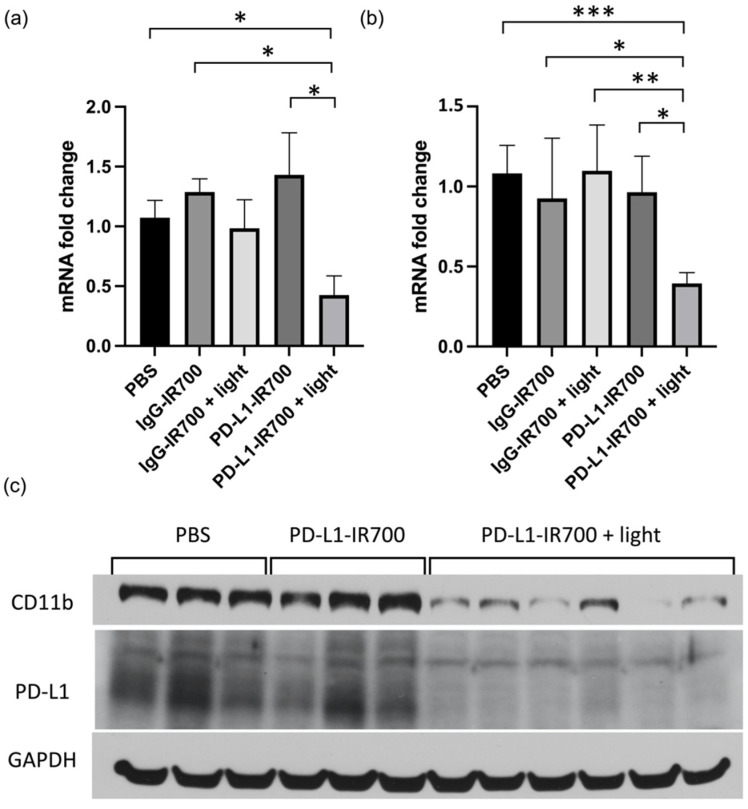
mRNA expression levels in orthotopic tumors 3 weeks post NIR-PIT for PD-L1 (**a**) and CD11b (**b**) (PBS *n* = 6, IgG-IR700 *n* = 3, IgG-IR700 + light *n* = 5, PD-L1 *n* = 8, PD-L1 + light *n* = 9) (* *p* < 0.05, ** *p* < 0.01, *** *p* < 0.005). PD-L1 and CD11b immunoblots of ID8-VEGF-Defb29 tumor extracts after PBS (*n* = 3), PD-L1-IR700 (*n* = 3), and PD-L1 + light (*n* = 6) treatment (**c**). GAPDH was used as loading control.

Three weeks post treatment, tumors were excised and extracted for RT-PCR and immunoblot analysis. No differences in CD11b were observed in the PBS and IgG-IR700 groups, with or without NIR-PIT. A significant decrease of CD11b was observed in the PD-L1-IR700 with NIR-PIT. The decrease was measured at the mRNA levels, as well as protein levels (Figure 6). PD-L1 levels also decreased in the PD-L1-IR700 NIR-PIT group. No differences were observed at three weeks post treatment in AST, ALT, and BUN plasma levels between the groups (data not shown).

## 4. Discussion

PD-L1 is expressed in ID8-Defb29-VEGF tumors, with higher levels detected in orthotopic tumors as compared to subcutaneous tumors, highlighting the importance of the tumor microenvironment in the expression of PD-L1. Other studies have shown differences in immune markers between subcutaneous and orthotopic tumors. The number of T cells was higher in orthotopic colon tumors as compared to subcutaneous tumors. This difference was associated with enhanced levels of pro-inflammatory cytokines, including IL-2 and IFN-γ, which resulted in a higher sensitivity of the orthotopic tumors to immune checkpoint inhibitors [28]. Differences in macrophage types and in CD4/CD8 ratios were observed in lung cancer models [29]. Since we did not observe any differences in F4/80 between orthotopic and subcutaneous tumors, the PD-L1 differences observed here were not due to changes in macrophage number.

We detected expression of CD68 in ID8-Defb29-VEGF cells. CD68, a highly glycosylated type I transmembrane glycoprotein, mainly localized within the endosomal compartment, is selective for the monocyte/macrophage lineage and often used as a monocyte/macrophage specific marker. Previous studies have reported the expression of CD68 by other cells, including fibroblasts, endothelial cells, and tumor cells [30,31]. In our model, because the expression of CD68 was detected in cancer cells and did not appear to be restricted to macrophages, it was not considered a TAM marker.

We confirmed the efficacy of PD-L1 targeted NIR-PIT in ovarian cancer cells following PD-L1 induction with IFN-γ. In macrophages, induction with IFN-γ was not necessary to cause significant cell killing. We observed that following treatment with IFN-γ, RAW264.7 cells were highly sensitive to light shining, even with non-targeted IgG-IR700. The high sensitivity to NIR-PIT could be due to macrophage activation induced by IFN-γ treatment and the resulting higher uptake of IR700 with or without specific targeting [32].

Photodynamic therapy (PDT) and NIR-PIT have been previously applied to ovarian cancer. EGFR-targeted NIR-PIT was tested in ovarian tumor cluster spheroids grown either under flow or static conditions and was found to be equally effective in both settings [33]. The efficacy of PIT after NIR irradiation in terms of cell killing and tumor shrinkage was demonstrated in preclinical models of ovarian cancer [34,35]. One study, performed using a subcutaneous or peritoneal SKOV3 ovarian cancer model targeting HER2 in immunodeficient mice, confirmed the anti-tumor effects of NIR-PIT from a reduction in tumor volume [34]. The beta-D-galactose receptor was targeted using galactosyl serum albumin in an SHIN3-disseminated peritoneal ovarian cancer model [35]. Repeated NIR-PIT using GSA-IR700 achieved efficient antitumor effects [35]. In another study, C225 antibody targeting EGFR combined with benzoporphyrin derivate monoacid A (BPD)-PDT showed efficacy in an OVCAR5 model of ovarian cancer [36]. BPD-PDT induced cancer cell apoptosis via a mitochondrially targeted release of cytochrome c, triggering activation of different caspases. To the best of our knowledge, PD-L1-targeted NIR-PIT has not been applied to ovarian cancer. A previous study performed with a human lung adenocarcinoma xenograft inoculated in immune-deficient mice has shown the potential of using PD-L1 targeted NIR-PIT [9]. In that study, a PD-L1-expressing human lung cancer cell line, H441, was treated in vitro and in vivo with avelumab-IR700 and NIR light. Tumor growth was reduced and survival was significantly prolonged in the NIR-PIT-treated group as compared to the different controls. The treatment protocol for the subcutaneous tumors consisted of a single injection of avelumab-IR700 and two light exposures [9]. 

NIR-PIT has direct cytotoxicity on cells targeted by the IR700–antibody complex combined with light exposure, but it can also have indirect effects on the tumor microenvironment. It has been shown that targeting Treg with CD25(Fab’)_2_-IR700 induced a selective depletion of intra-tumoral Treg, causing IFN-γ-dependent acute tumor vessel regression. Upon selective depletion of Treg, IFN-γ induced in CD8T cells and NK cells directly targeted endothelial cells to cause rapid vessel regression, intratumoral ischemia, and necrosis or apoptosis of tumor cells [37]. Further studies are needed to explore the indirect effects of targeting PD-L1 in our tumor model, including the effects on immune cell recruitment in the tumor post therapy at early and late time points.

PD-L1 expression by CAFs has been observed in non-small cell lung carcinoma [38] and in breast cancer [39]. Moreover, in ascites originating from ID8 tumor, 20–40% of CD45+ immune cells expressed PD-L1 [40]. High PD-L1 expression was also observed in TAMs, MDSCs, and dendritic cells. PD-L1 was also detected on ID8 cancer cells [40]. Human tissues analysis showed detectable levels of PD-L1 on ovarian cancer cells in 35% of patients examined. PD-L1-positive dendritic cells and TAMs were also detected in the ovarian cancer tumor microenvironment [40]. The PD-L1 NIR-PIT studies performed here would have targeted all PD-L1 expressing cells, including cancer and stromal cells, which would have contributed to the changes observed post treatment.

We observed significant toxicity with PD-L1-IR700 NIR-PIT, as 55% of the mice treated died within 24 h post treatment, although treatment with PD-L1 antibody alone had no toxicity. No other NIR-PIT-treated group presented any similar toxicity. Immune checkpoint inhibitors can induce immune related adverse events (irAEs) which can affect any organ, and range from asymptomatic to fulminant [41]. In the clinic, irAEs take from several days to weeks to develop, including pneumonitis (focal or diffuse inflammation of the lung parenchyma, which occurs in 1% of patients), colitis/diarrhea (most common), hepatitis, dermatologic toxicities, and endocrinopathies. Toxicity after PD-1/PD-L1 targeting has been previously described in a 4T1 breast tumor model implanted in Balb/C mice [27]. A mortality of >85% was observed in mice treated with repeated injections of either anti-PD-1 or anti-PD-L1. The toxicity was not observed in non-tumor-bearing mice, in different tumor-bearing mice, or in mice treated with other antibodies [27]. The fatal hypersensitivity reactions observed were due to an accumulation of neutrophils within the lungs of the mice as well as an increase in IgG1 antibodies in the serum [27]. Similar events may have occurred in our model following PD-L1 NIR-PIT, but not PD-L1 alone, with the PD-1/PD-L1 axis potentially exacerbating a hypersensitivity reaction. The combination of PD-L1-IR700 and the light irradiation induced toxicity in 55% of the mice, but the remaining 45% remained unaffected. In a study targeting EGFR and HER2 in a bladder cancer model in immunodeficient mice, increases of ALT, AST, and BUN were observed, similarly to our results [42], but there was no evidence of damage in H&E sections of the liver and kidneys. Four out of eleven treated mice died within 48 h of light exposure [42], indicative of a strong systemic inflammatory response due to massive tumor necrosis and tumor lysis syndrome. Our toxicity studies showed some limited damage in the liver and kidneys directly linked to light exposure, highlighting the need for light protection of normal tissue to minimize collateral phototoxicity. One of the five treated mice in our toxicity studies showed significant damage to the lungs that was not due to light exposure, with the highest levels of AST and ALT, but not BUN. Accumulation of immune cells could be visualized, indicative of a hypersensitivity reaction that was only observed with the combination of PDL1-IR700 injection and light exposure. Future studies investigating the different outcomes in different mice will be needed to expand our understanding of these syndromes and of the immune-related adverse events that can occur with PD-L1 targeted therapy.

## 5. Conclusions

Overall, our findings support further preclinical safety assessments to evaluate NIR-PIT as a potential therapeutic option for ovarian cancer. The efficacy of NIR-PIT depends on the ability to deliver NIR light to the tissue specifically targeted by the IR700-antibody complex. In the translational setting, PD-L1 NIR-PIT could be applied intraoperatively during debulking of ovarian cancer or using an endoscopic approach to target peritoneally disseminated disease, as shown in a model of Her2+ gastric carcinoma [8]. Combining external and interstitial light sources could improve treatment efficacy, as shown in a human squamous carcinoma model [6]. In the NIR-PIT clinical trial in head and neck cancer patients, combining external and interstitial light is the standard therapy. While the procedure is invasive due to the placement of an interstitial catheter, it improves tumor response to therapy [6]. In the context of ovarian cancer, residual tumor and peritoneal metastases could be exposed to NIR light during surgery. Additionally, other cancer-specific or TAM-associated targets could be used for NIR-PIT to improve treatment outcome for ovarian cancer patients, as optimal surgical tumor debulking significantly improves patient survival compared to suboptimal debulking, causing widespread lesions located throughout the abdomen and often preventing complete removal of the tumor. PD-L1-targeted NIR-PIT can fill an important niche in ovarian cancer treatment in combination with conventional treatments to improve outcome.

## Data Availability

The data presented in this study are available on request from the corresponding author.

## Supplementary Materials

The following supporting information can be downloaded at: https://www.mdpi.com/article/10.3390/cancers14030619/s1, Table S1: Primers used for the RT-PCR analysis, Figure S1: CD68 expression in ID8-Defb29-VEGF and RAW264.7 cells, Figure S2: RAW264 viability with no light exposure, and with IFNg pre-treatment, Figure S3: Representative example of the light irradiation setup and NIR images of the corresponding tumor bearing mouse before and after photoirradiation, Figure S4: Plasma levels of ALT, AST and BUN in ID8-Defb29-VEGF tumor bearing mice, Figure S5: H&E stained sections of heart from tumor bearing mice, Figure S6: H&E stained sections of kidney from tumor bearing mice, Figure S7: H&E stained sections of liver from tumor bearing mice, Figure S8: H&E stained sections of inflated lungs from tumor bearing mice, Figure S9: H&E stained sections of inflated lungs from a tumor bearing mouse treated with PDL1-IR700 + light showing visible damage, Figure S10: Original blots.

## References

[1] Siegel R.L., Miller K.D., Jemal A. (2016). Cancer statistics. CA Cancer J. Clin..

[2] Stanwell P., Russell P., Carter J., Pather S., Heintze S.C. (2008). Mountford, Evaluation of ovarian tumors by proton magnetic resonance spectroscopy at three Tesla. Investig. Radiol..

[3] Lheureux S., Braunstein M., Oza A.M. (2019). Epithelial ovarian cancer: Evolution of management in the era of precision medicine. CA Cancer J. Clin..

[4] Riester M., Wei W., Waldron L., Culhane A.C., Trippa L., Oliva E., Kim S.H., Michor F., Huttenhower C., Parmigiani G. (2014). Risk prediction for late-stage ovarian cancer by meta-analysis of 1525 patient samples. J. Natl. Cancer Inst..

[5] Kobayashi H., Furusawa A., Rosenberg A., Choyke P.L. (2021). Near-infrared photoimmunotherapy of cancer: A new approach that kills cancer cells and enhances anti-cancer host immunity. Int. Immunol..

[6] Maruoka Y., Nagaya T., Sato K., Ogata F., Okuyama S., Choyke P.L., Kobayashi H. (2018). Near Infrared Photoimmunotherapy with Combined Exposure of External and Interstitial Light Sources. Mol. Pharm..

[7] Ogawa M., Tomita Y., Nakamura Y., Lee M.J., Lee S., Tomita S., Nagaya T., Sato K., Yamauchi T., Iwai H. (2017). Immunogenic cancer cell death selectively induced by near infrared photoimmunotherapy initiates host tumor immunity. Oncotarget.

[8] Nagaya T., Okuyama S., Ogata F., Maruoka Y., Choyke P.L., Kobayashi H. (2018). Endoscopic near infrared photoimmunotherapy using a fiber optic diffuser for peritoneal dissemination of gastric cancer. Cancer Sci..

[9] Nagaya T., Nakamura Y., Sato K., Harada T., Choyke P.L., Hodge J.W., Schlom J., Kobayashi H. (2017). Near infrared photoimmunotherapy with avelumab, an anti-programmed death-ligand 1 (PD-L1) antibody. Oncotarget.

[10] Jin J., Krishnamachary B., Mironchik Y., Kobayashi H., Bhujwalla Z.M. (2016). Phototheranostics of CD44-positive cell populations in triple negative breast cancer. Sci. Rep..

[11] Cognetti D.M., Johnson J.M., Curry J.M., Kochuparambil S.T., McDonald D., Mott F., Fidler M.J., Stenson K., Vasan N.R., Razaq M.A. (2021). Phase 1/2a, open-label, multicenter study of RM-1929 photoimmunotherapy in patients with locoregional, recurrent head and neck squamous cell carcinoma. Head Neck.

[12] Tahara M., Okano S., Enokida T., Ueda Y., Fujisawa T., Shinozaki T., Tomioka T., Okano W., Biel M.A., Ishida K. (2021). A phase I, single-center, open-label study of RM-1929 photoimmunotherapy in Japanese patients with recurrent head and neck squamous cell carcinoma. Int. J. Clin. Oncol..

[13] Gillenwater A.M., Cognetti D., Johnson J.M., Curry J., Kochuparambil S.T., McDonald D., Fidler M.J., Stenson K., Vasan N., Razaq M. (2018). RM-1929 photo-immunotherapy in patients with recurrent head and neck cancer: Results of a multicenter phase 2a open-label clinical trial. J. Clin. Oncol..

[14] Cognetti D.M., Johnson J.M., Curry J.M., Mott F., Kochuparambil S.T., McDonald D., Fidler M.J., Stenson K., Vasan N.R., Razaq M. (2019). Results of a phase 2a, multicenter, open-label, study of RM-1929 photoimmunotherapy (PIT) in patients with locoregional, recurrent head and neck squamous cell carcinoma (rHNSCC). J. Clin. Oncol..

[15] Kobayashi H., Choyke P.L. (2019). Near-Infrared Photoimmunotherapy of Cancer. Acc. Chem. Res..

[16] Nath S., Saad M.A., Pigula M., Swain J.W.R., Hasan T. (2019). Photoimmunotherapy of Ovarian Cancer: A Unique Niche in the Management of Advanced Disease. Cancers.

[17] Gaillard S.L., Coleman R.L. (2019). Identifying markers of immune response in ovarian cancer: Does PD-L1 expression meet the mark?. Ann. Oncol..

[18] Baci D., Bosi A., Gallazzi M., Rizzi M., Noonan D.M., Poggi A., Bruno A., Mortara L. (2020). The Ovarian Cancer Tumor Immune Microenvironment (TIME) as Target for Therapy: A Focus on Innate Immunity Cells as Therapeutic Effectors. Int. J. Mol. Sci..

[19] Webb J.R., Milne K., Kroeger D.R., Nelson B.H. (2016). PD-L1 expression is associated with tumor-infiltrating T cells and favorable prognosis in high-grade serous ovarian cancer. Gynecol. Oncol..

[20] Parvathareddy S.K., Siraj A.K., Al-Badawi I.A., Tulbah A., Al-Dayel F., Al-Kuraya K.S. (2021). Differential expression of PD-L1 between primary and metastatic epithelial ovarian cancer and its clinico-pathological correlation. Sci. Rep..

[21] Nowak M., Klink M. (2020). The Role of Tumor-Associated Macrophages in the Progression and Chemoresistance of Ovarian Cancer. Cells.

[22] Roby K.F., Taylor C.C., Sweetwood J.P., Cheng Y., Pace J.L., Tawfik O., Persons D.L., Smith P.G., Terranova P.F. (2000). Development of a syngeneic mouse model for events related to ovarian cancer. Carcinogenesis.

[23] Zhang L., Yang N., Garcia J.R., Mohamed A., Benencia F., Rubin S.C., Allman D., Coukos G. (2002). Generation of a syngeneic mouse model to study the effects of vascular endothelial growth factor in ovarian carcinoma. Am. J. Pathol..

[24] Conejo-Garcia J.R., Benencia F., Courreges M.C., Kang E., Mohamed-Hadley A., Buckanovich R.J., Holtz D.O., Jenkins A., Na H., Zhang L. (2004). Tumor-infiltrating dendritic cell precursors recruited by a beta-defensin contribute to vasculogenesis under the influence of Vegf-A. Nat. Med..

[25] Bharti S.K., Wildes F., Hung C.F., Wu T.C., Bhujwalla Z.M., Penet M.F. (2017). Metabolomic characterization of experimental ovarian cancer ascitic fluid. Metabolomics.

[26] Penet M.F., Krishnamachary B., Wildes F., Mironchik Y., Mezzanzanica D., Podo F., de Reggi M., Gharib B., Bhujwalla Z.M. (2016). Effect of Pantethine on Ovarian Tumor Progression and Choline Metabolism. Front. Oncol..

[27] Mall C., Sckisel G.D., Proia D.A., Mirsoian A., Grossenbacher S.K., Pai C.S., Chen M., Monjazeb A.M., Kelly K., Blazar B.R. (2016). Repeated PD-1/PD-L1 monoclonal antibody administration induces fatal xenogeneic hypersensitivity reactions in a murine model of breast cancer. Oncoimmunology.

[28] Zhao X., Li L., Starr T.K., Subramanian S. (2017). Tumor location impacts immune response in mouse models of colon cancer. Oncotarget.

[29] Li H.Y., McSharry M., Bullock B., Nguyen T.T., Kwak J., Poczobutt J.M., Sippel T.R., Heasley L.E., Weiser-Evans M.C., Clambey E.T. (2017). The Tumor Microenvironment Regulates Sensitivity of Murine Lung Tumors to PD-1/PD-L1 Antibody Blockade. Cancer Immunol. Res..

[30] Gottfried E., Kunz-Schughart L.A., Weber A., Rehli M., Peuker A., Muller A., Kastenberger M., Brockhoff G., Andreesen R., Kreutz M. (2008). Expression of CD68 in non-myeloid cell types. Scand. J. Immunol..

[31] Gloghini A., Rizzo A., Zanette I., Canal B., Rupolo G., Bassi P., Carbone A. (1995). KP1/CD68 expression in malignant neoplasms including lymphomas, sarcomas, and carcinomas. Am. J. Clin. Pathol..

[32] Su X., Yu Y., Zhong Y., Giannopoulou E.G., Hu X., Liu H., Cross J.R., Ratsch G., Rice C.M., Ivashkiv L.B. (2015). Interferon-gamma regulates cellular metabolism and mRNA translation to potentiate macrophage activation. Nat. Immunol..

[33] Nath S., Pigula M., Khan A.P., Hanna W., Ruhi M.K., Dehkordy F.M., Pushpavanam K., Rege K., Moore K., Tsujita Y. (2020). Flow-induced Shear Stress Confers Resistance to Carboplatin in an Adherent Three-Dimensional Model for Ovarian Cancer: A Role for EGFR-Targeted Photoimmunotherapy Informed by Physical Stress. J. Clin. Med..

[34] Sato K., Hanaoka H., Watanabe R., Nakajima T., Choyke P.L., Kobayashi H. (2015). Near infrared photoimmunotherapy in the treatment of disseminated peritoneal ovarian cancer. Mol. Cancer Ther..

[35] Harada T., Nakamura Y., Sato K., Nagaya T., Okuyama S., Ogata F., Choyke P.L., Kobayashi H. (2016). Near-infrared photoimmunotherapy with galactosyl serum albumin in a model of diffuse peritoneal disseminated ovarian cancer. Oncotarget.

[36] del Carmen M.G., Rizvi I., Chang Y., Moor A.C., Oliva E., Sherwood M., Pogue B., Hasan T. (2005). Synergism of epidermal growth factor receptor-targeted immunotherapy with photodynamic treatment of ovarian cancer in vivo. J. Natl. Cancer Inst..

[37] Kurebayashi Y., Olkowski C.P., Lane K.C., Vasalatiy O.V., Xu B.C., Okada R., Furusawa A., Choyke P.L., Kobayashi H., Sato N. (2021). Rapid Depletion of Intratumoral Regulatory T Cells Induces Synchronized CD8 T- and NK-cell Activation and IFNgamma-Dependent Tumor Vessel Regression. Cancer Res..

[38] Teramoto K., Igarashi T., Kataoka Y., Ishida M., Hanaoka J., Sumimoto H., Daigo Y. (2019). Clinical significance of PD-L1-positive cancer-associated fibroblasts in pN0M0 non-small cell lung cancer. Lung Cancer.

[39] Yoshikawa K., Ishida M., Yanai H., Tsuta K., Sekimoto M., Sugie T. (2021). Prognostic significance of PD-L1-positive cancer-associated fibroblasts in patients with triple-negative breast cancer. BMC Cancer.

[40] Lin H., Wei S., Hurt E.M., Green M.D., Zhao L., Vatan L., Szeliga W., Herbst R., Harms P.W., Fecher L.A. (2018). Host expression of PD-L1 determines efficacy of PD-L1 pathway blockade-mediated tumor regression. J. Clin. Investig..

[41] Wang D.Y., Johnson D.B., Davis E.J. (2018). Toxicities Associated with PD-1/PD-L1 Blockade. Cancer J..

[42] Siddiqui M.R., Railkar R., Sanford T., Crooks D.R., Eckhaus M.A., Haines D., Choyke P.L., Kobayashi H., Agarwal P.K. (2019). Targeting Epidermal Growth Factor Receptor (EGFR) and Human Epidermal Growth Factor Receptor 2 (HER2) Expressing Bladder Cancer Using Combination Photoimmunotherapy (PIT). Sci. Rep..

