# Supplementary material for: PD-L1 near Infrared Photoimmunotherapy of Ovarian Cancer Model
Source: Cancers (Basel). 2022 Jan 26;14(3):619. doi: 10.3390/cancers14030619 (PMC8833482; doi:10.3390/cancers14030619)
Supplement: Supplementary file 1 [file cancers-14-00619-s001.zip › cancers-1575058-supplementary.pdf]

cancers

MDPI

Supplementary Materials: PD-L1 near Infrared Photoimmunotherapy of Ovarian Cancer Model

Jiefu Jin, Ishwarya Sivakumar, Yelena Mironchik, Balaji Krishnamachary, Flonné Wildes, James D. Barnett, Chien-Fu Hung, Sridhar Nimmagadda, Hisataka Kobayashi, Zaver M. Bhujwalla and Marie-France Penet

Supplementary Table S1. Primers used for the RT-PCR analysis.

	Gene of Interest	GeneBank ID	Sequence	Nucleotide position	Product size (base pairs)	Source
1	Mouse PD-L1	AF233517.1	Mm_PD-L1 Fwd-5'-tgccaaaggaccagcttttg -3'	276	149 bp	Primer 3 Plus
			Mm_PD-L1 Rev-5'-ttttgcggtatggggcattg -3'	425		
4	Mouse CD11b	NM_001082960	Mm_CD11b_Fwd -5'-TACGTAATTGGGGTGGGAA-3'	846	167 BP	Primer 3 Plus
			Mm_CD11b_Rev -5'-GTGCCCTCAATTGCAAAGAT-3'	1013		
5	Mouse CD68	NM_001291058.1	Mm_CD68_Fwd: ACTTCGGGCCATGTTTCTCT	835	138 bp	Volat FE et al., Diabetes. 2012. 61:2796-806
			Mm_CD68_Rev: GCTGGTAGGTTGATTGTCGT	973		
6	Mouse F4/80	XM_006523602	Mm_F4/80#2-Fwd-5'-AACATGCAACCTGCCACAAC-3'	131	137 bp	Primer 3 Plus
			Mm_F4/80#2-Rev-5'-TGAATTCCTGGAGCACTCATCC-3'	267		
7	18s rRNA		18S rRNA fwd-5'-CGGCGACGACCCATTCGAAC–3'			Beacon Designer
			18s rRNA Rev-5'-GAATCGAACCCTGATTCCCCGTC-3'			

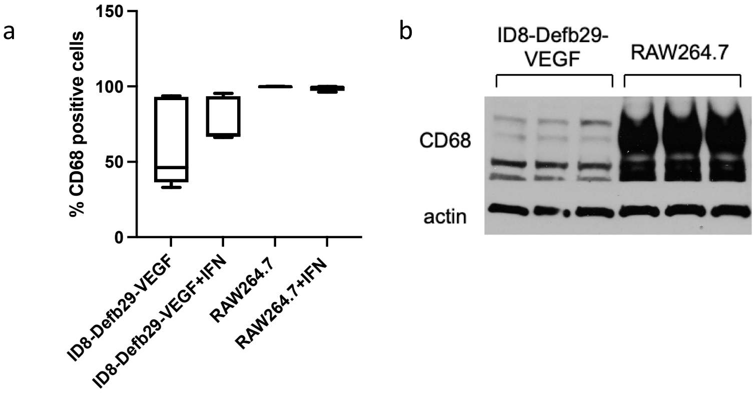

Supplementary Figure S1. CD68 intracellular expression in ID8-Defb29-VEGF (n=6) and RAW264.7 (n=5) cells analyzed by flow cytometry (a). CD68 expression levels in cell extracts analyzed by immunoblots (b). Actin was used as loading control.

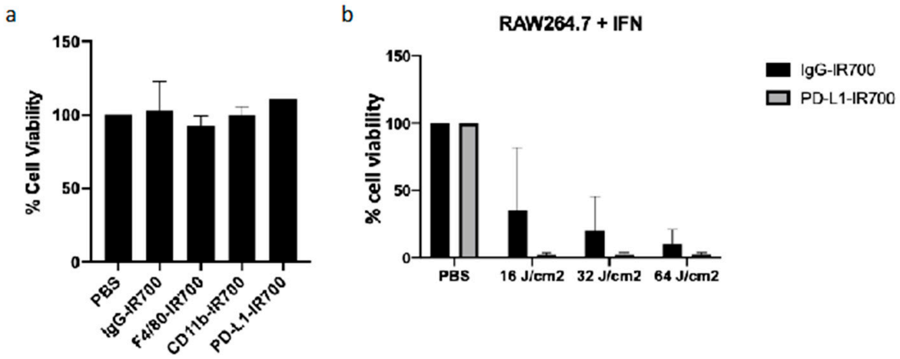

Supplementary Figure S2. Cell viability of RAW264 treated with PBS, or IR700 complex probes for 24hrs, with no light exposure (a). Cell viability of RAW264 pre-treated with IFNγ, treated for 24hrs with either IgG-IR700 or PD-L1-IR700 and exposed to different doses of irradiation (b).

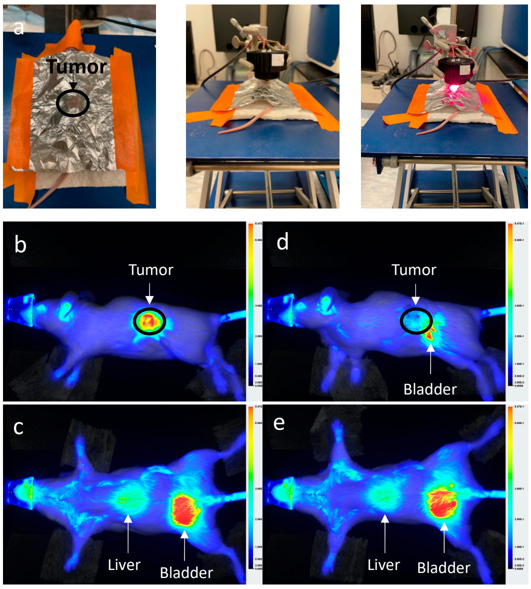

Supplementary Figure S3. Representative example of the light irradiation setup (a). Orthotopic tumor bearing mouse positioned on its flank, with the tumor on the upper side. Aluminum foil is used to cover the body with a small opening cut around the tumor area, to expose only the tumor to light. NIR images of the corresponding tumor bearing mouse before (b-c) and after (d-e) photoirradiation, showing the decreased signal in the tumor, but no reduction of light intensity in the liver and bladder.

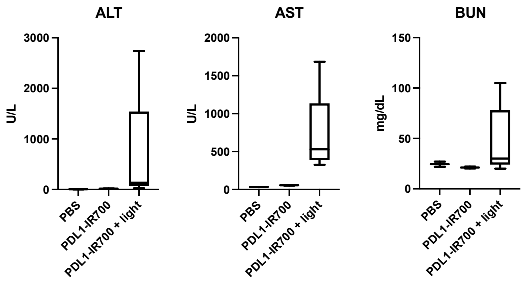

Supplementary Figure S4. Plasma levels of ALT, AST and BUN in ID8-Defb29-VEGF tumor bearing mice (Means +/- SD are shown; PBS n=2, PDL1-IR700 n=2, PDL1-IR700 + light n=5).

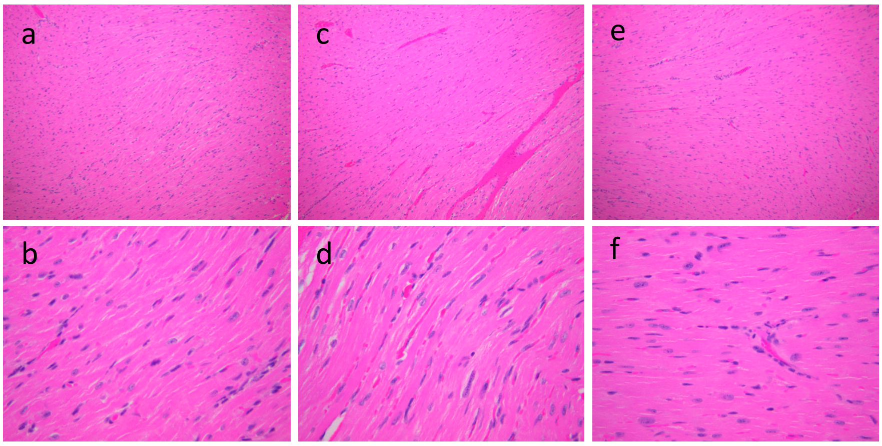

Supplementary Figure S5. H&E stained sections of heart from tumor bearing mice: control (a-b), PDL1-IR700 (c-d), or PDL1-IR700 + light (e-f) at magnification x10 (top row) and x40 (bottom row).

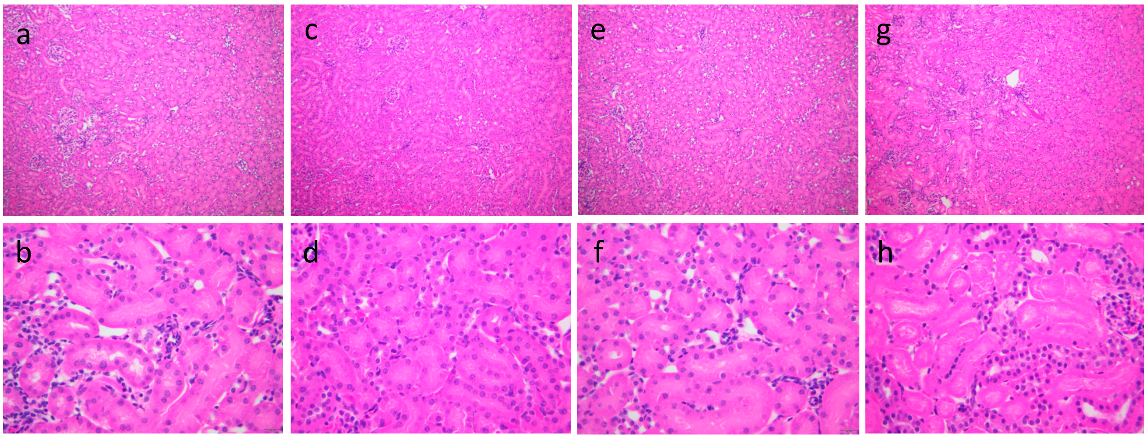

Supplementary Figure S6. H&E stained sections of kidney from tumor bearing mice: control (a-b), PDL1-IR700 (c-d), or PDL1-IR700 + light (no light side e-f; light side g-h) at magnification x10 (top row) and x40 (bottom row).

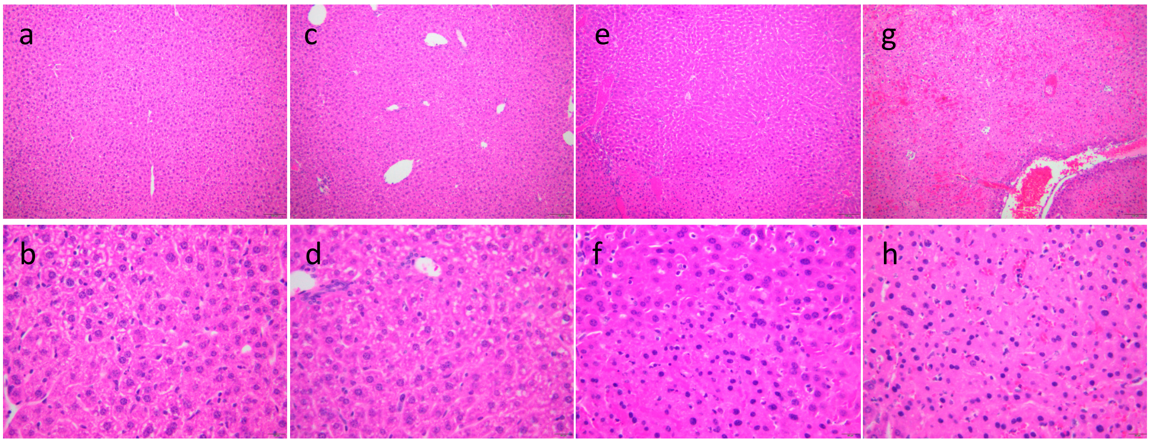

Supplementary Figure S7. H&E stained sections of liver from tumor bearing mice: control (a-b), PDL1-IR700 (c-d), or 2 PDL1-IR700 + light showing damage (e-h) at magnification x10 (top row) and x40 (bottom row).

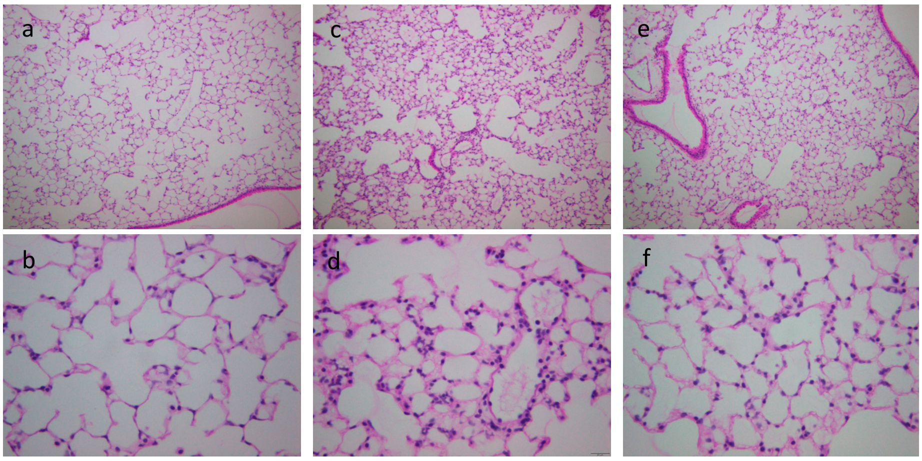

Supplementary Figure S8. H&E stained sections of inflated lungs from tumor bearing mice: control (a-b), PDL1-IR700 (c-d), or PDL1-IR700 + light with no visible damage (e-f) at magnification x10 (top row) and x40 (bottom row).

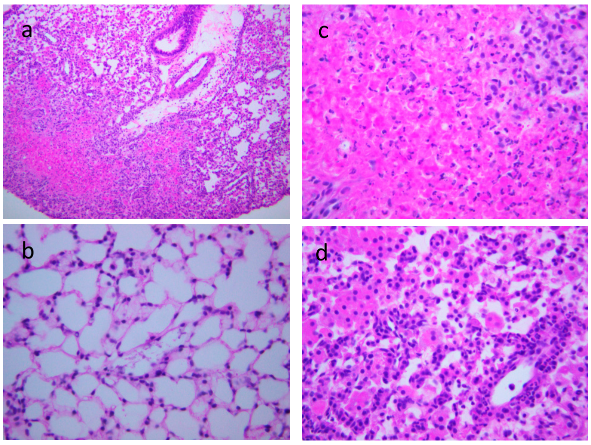

Supplementary Figure S9. H&E stained sections of inflated lungs from a tumor bearing mouse treated with PDL1-IR700 + light showing visible damage at magnification x10 (a) and x40 (b-d).
