# Supplementary figures and images for: PD-L1 near Infrared Photoimmunotherapy of Ovarian Cancer Model
Source: Cancers (Basel). 2022 Jan 26;14(3):619. doi: 10.3390/cancers14030619 (PMC8833482; doi:10.3390/cancers14030619)

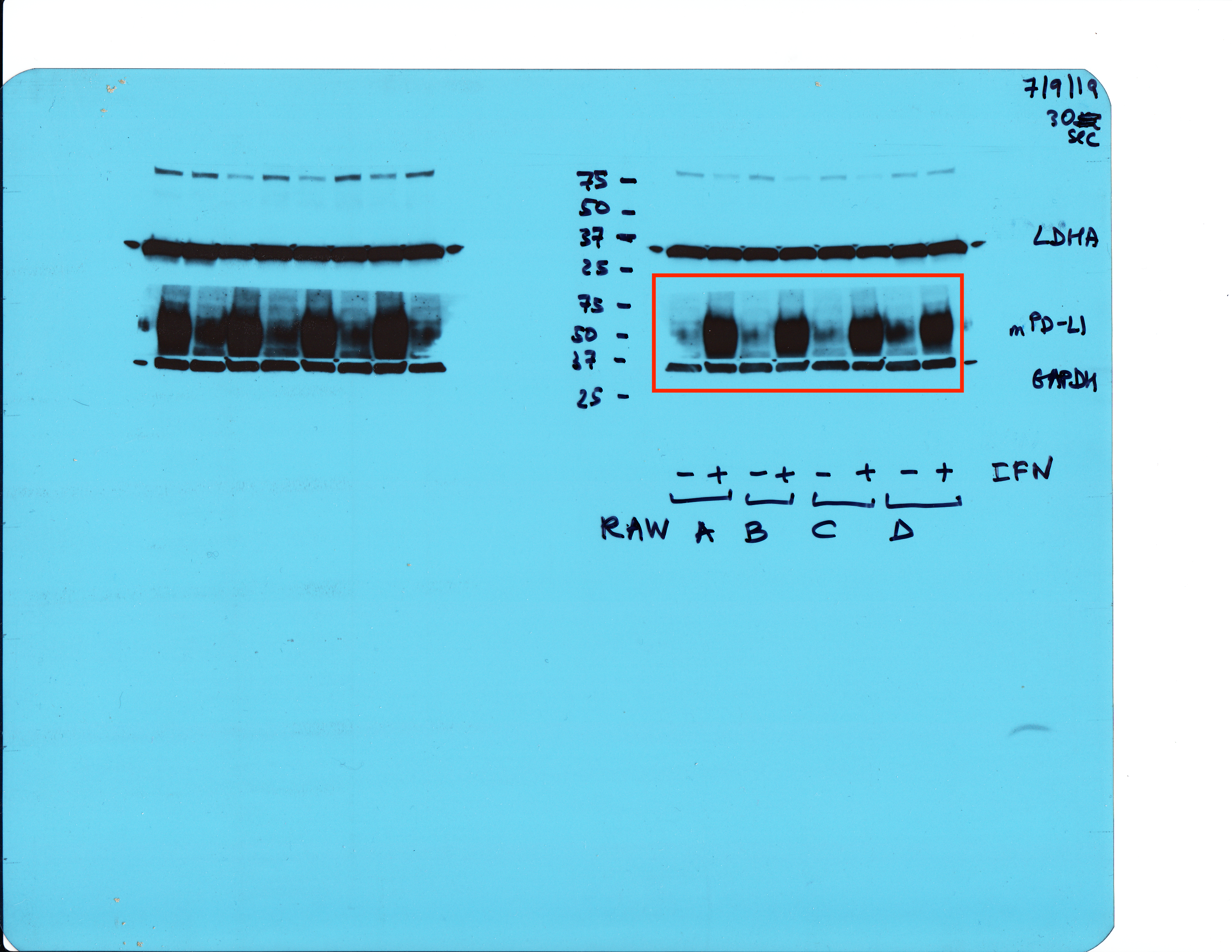

Supplement: Supplementary file 1 [file cancers-14-00619-s001.zip › cancers-1575058 -supplementary Figure S10 original blots/Figure2b_blot.tif]

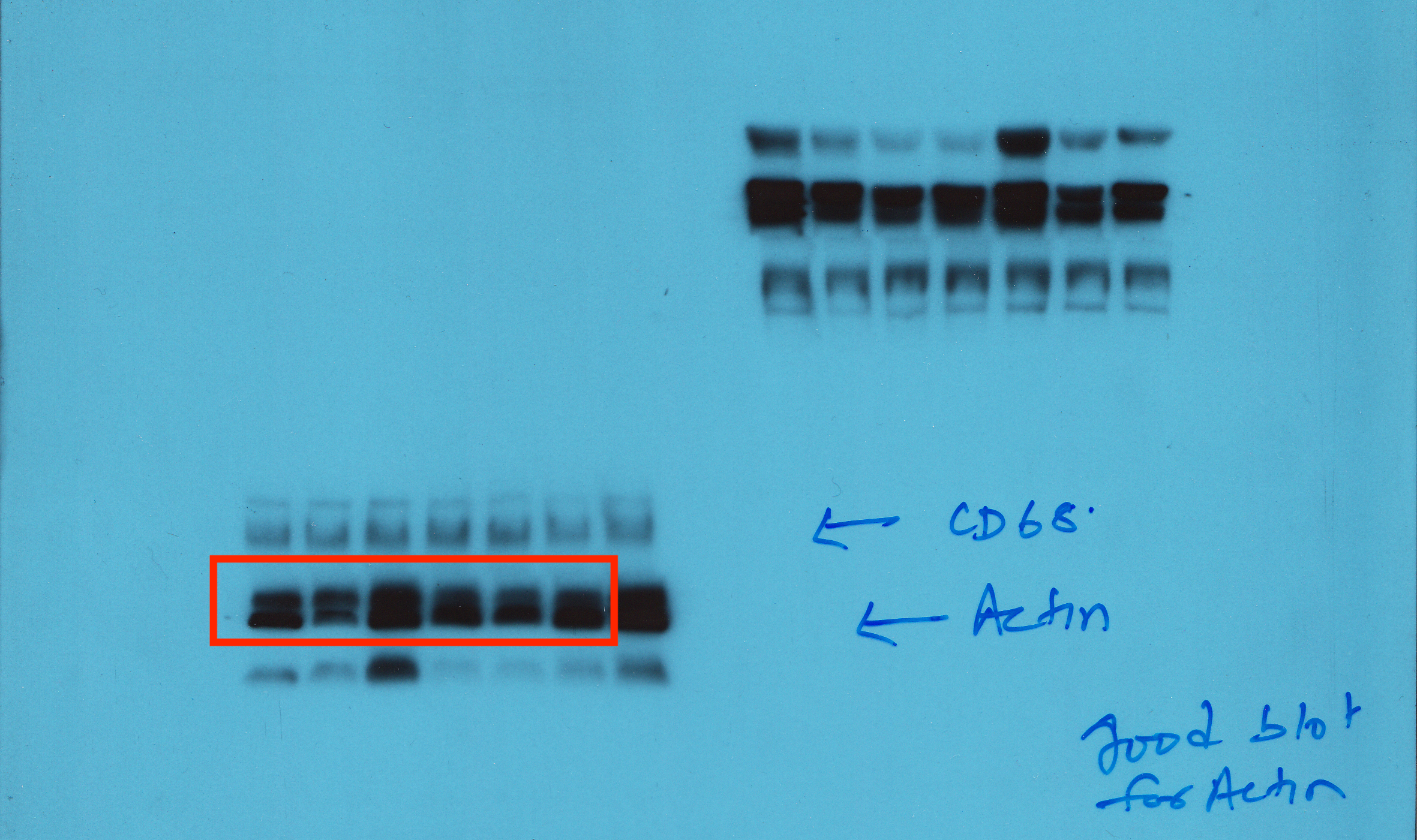

Supplement: Supplementary file 1 [file cancers-14-00619-s001.zip › cancers-1575058 -supplementary Figure S10 original blots/Figure4b_actin.tif]

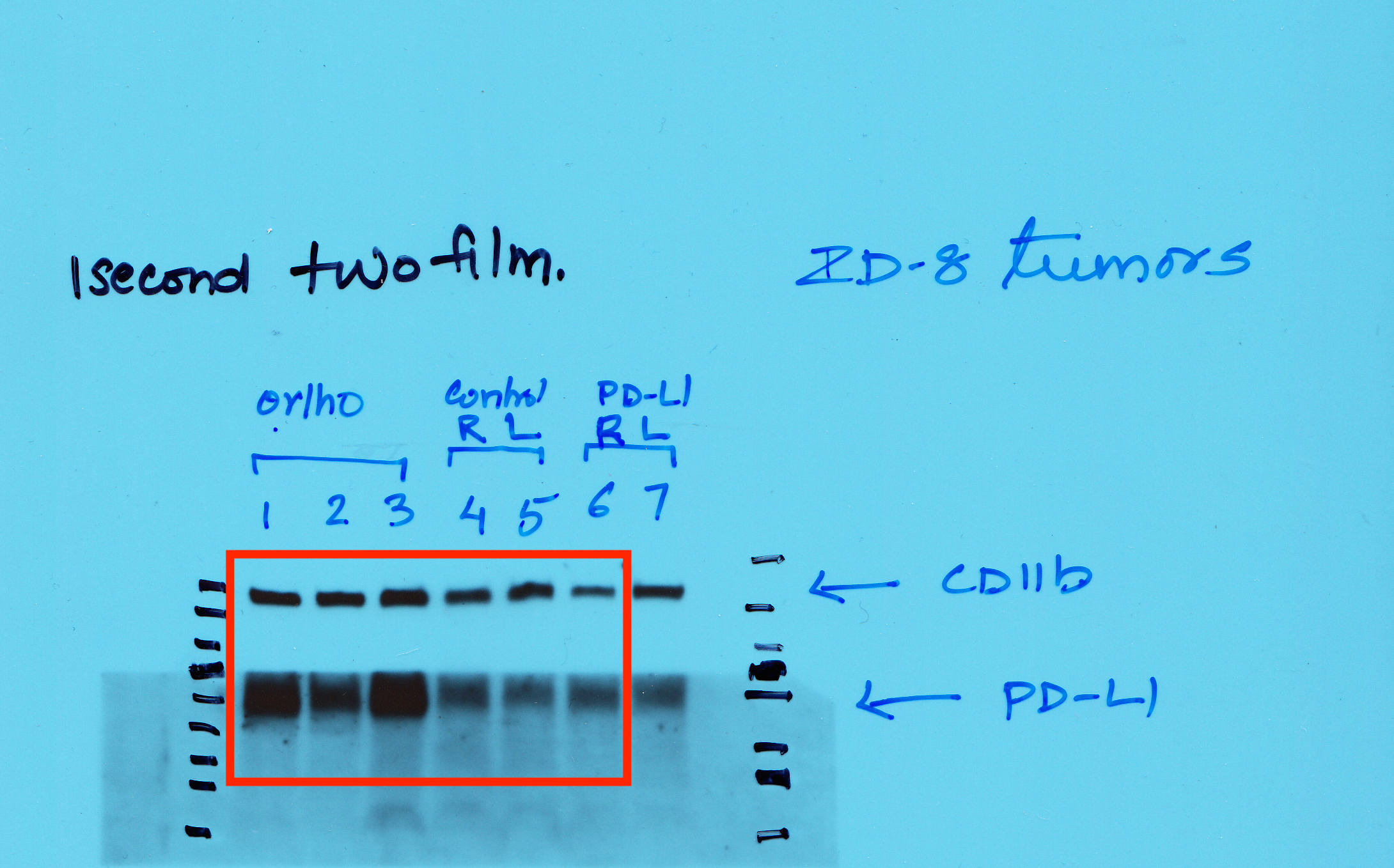

Supplement: Supplementary file 1 [file cancers-14-00619-s001.zip › cancers-1575058 -supplementary Figure S10 original blots/Figure4b_CD11b-PDl1.tif]

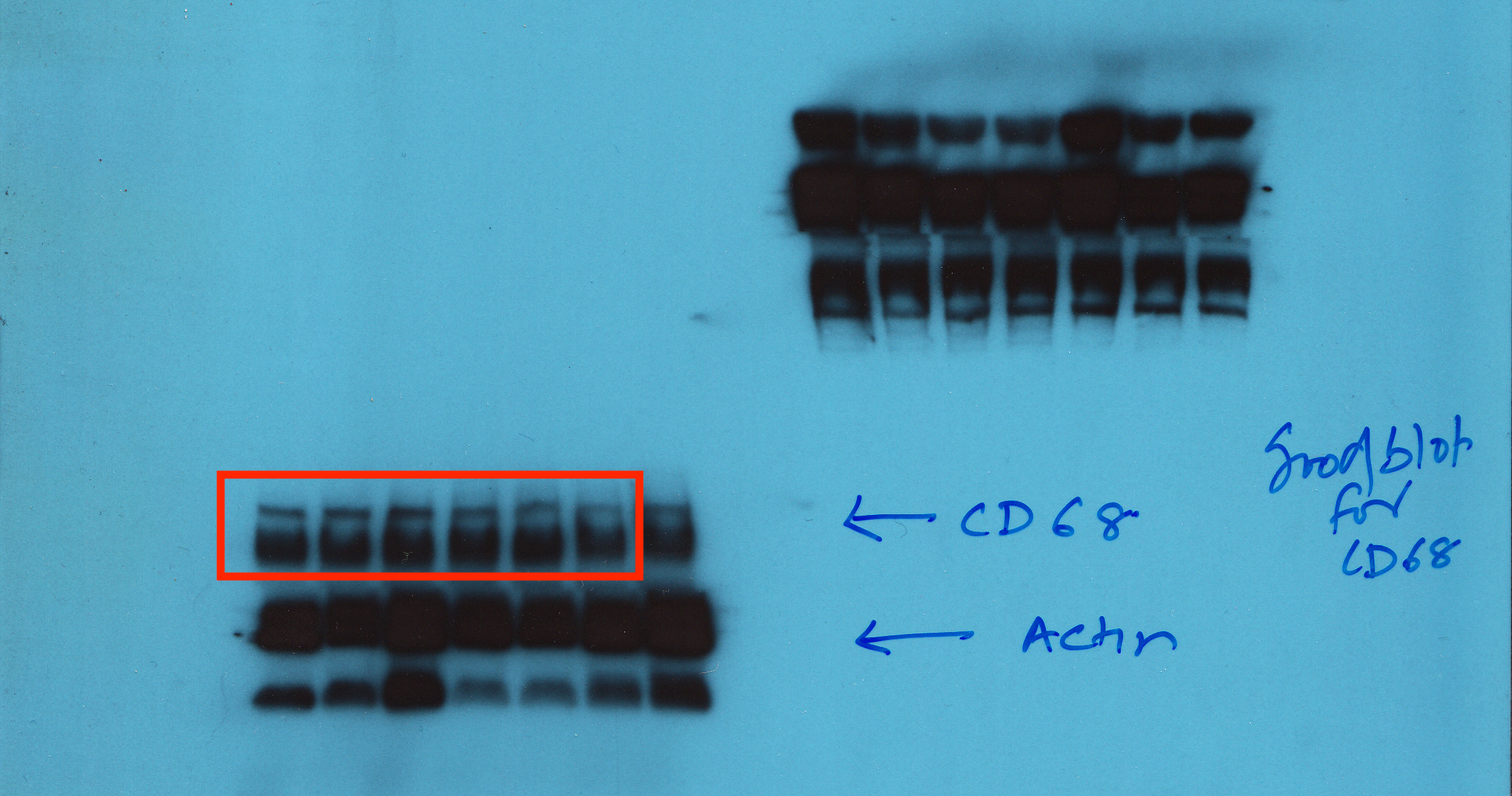

Supplement: Supplementary file 1 [file cancers-14-00619-s001.zip › cancers-1575058 -supplementary Figure S10 original blots/Figure4b_CD68.tif]

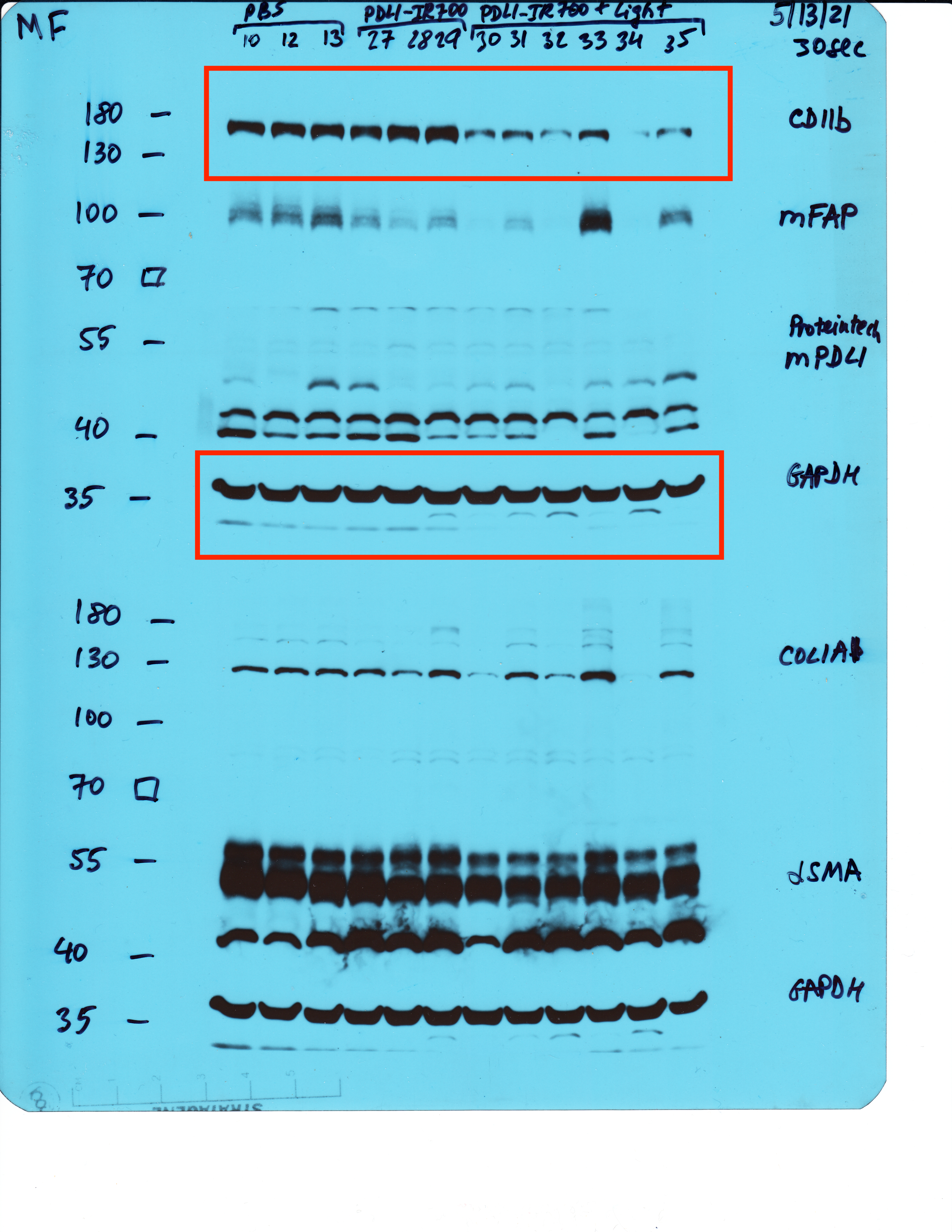

Supplement: Supplementary file 1 [file cancers-14-00619-s001.zip › cancers-1575058 -supplementary Figure S10 original blots/Figure6c_CD11b-GAPDH.tif]

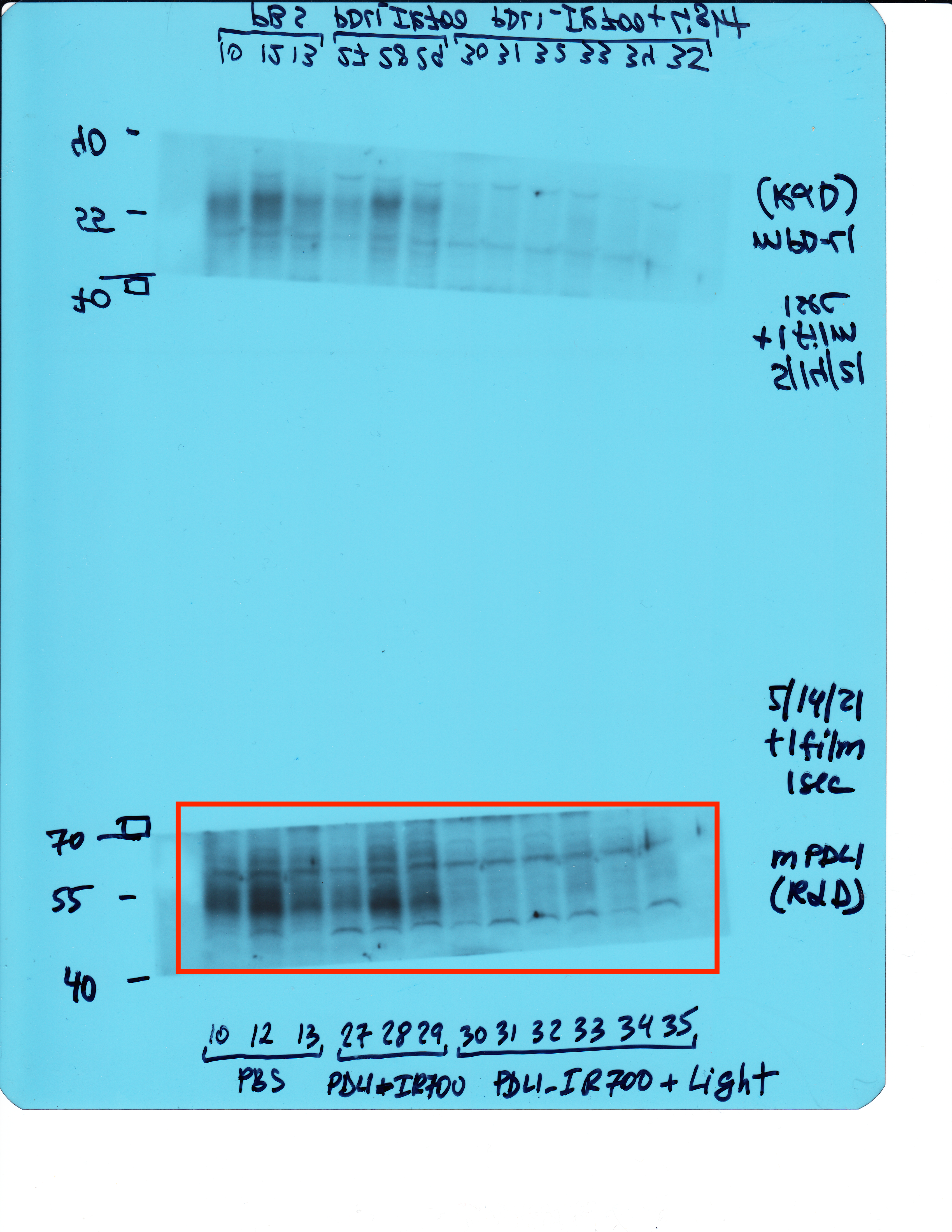

Supplement: Supplementary file 1 [file cancers-14-00619-s001.zip › cancers-1575058 -supplementary Figure S10 original blots/Figure6c_PDL1.tif]
